# Improved Superb Fairy-Wren Optimization Algorithm and Its Application

**DOI:** 10.3390/biomimetics11020093

**Published:** 2026-02-01

**Authors:** Yachao Cao, Hexuan Lv, Yanping Cui, Zhe Wu, Qiang Zhang

**Affiliations:** 1 School of Mechanical Engineering, Hebei University of Science and Technology, Shijiazhuang 050018, China; yachao.cao@hebust.edu.cn (Y.C.); 17734293309@163.com (H.L.); wuzhe@hebust.edu.cn (Z.W.); 2 Key Laboratory of Vehicle Transmission, China North Vehicle Research Institute, Beijing 100072, China; qiangzh36@gmail.com

**Keywords:** Superb Fairy-wren Optimization Algorithm, Improved Superb Fairy-wren Optimization Algorithm, Chebyshev chaotic map, adaptive weighting factor, Cauchy–Gaussian mutation, t-distribution perturbation

## Abstract

The Superb Fairy-wren Optimization Algorithm (SFOA) is a meta-heuristic algorithm inspired by the behavior of the superb fairy-wren. However, the conventional SFOA tends to converge to local optima and exhibits limited convergence accuracy when addressing complex optimization problems. To overcome these drawbacks, this study proposes an Improved Superb Fairy-wren Optimization Algorithm (ISFOA). The ISFOA incorporates four strategies—Chebyshev chaotic mapping, an adaptive weighting factor, Cauchy–Gaussian mutation, and t-distribution perturbation—to enhance the algorithm’s ability to balance global exploration and local exploitation. An ablation study using the CEC 2021 test suite was performed to evaluate the individual contribution of each strategy. Moreover, to comprehensively assess the performance of ISFOA, a comparative analysis was carried out against eight other meta-heuristic algorithms on both the CEC2005 and CEC2021 benchmark function sets. Additionally, the practical applicability of ISFOA was examined by comparing it with eight other optimization algorithms across seven engineering design problems. The comprehensive experimental results indicate that ISFOA outperforms the original SFOA and other compared algorithms in terms of robustness and convergence accuracy, thereby offering an efficient and reliable approach for solving complex optimization problems.

## 1. Introduction

The deepening process of global industrialization has led to increasingly large-scale and complex problems in areas such as industrial production, logistics management, and energy scheduling. Traditional optimization methods often face computational bottlenecks when addressing such complex, high-dimensional, nonlinear, and multi-constrained problems [1]. Metaheuristic optimization algorithms [2] are a class of intelligent optimization techniques that do not rely on specific mathematical properties of the objective function. Owing to their strong global search capability and high adaptability, they have shown significant promise in solving diverse complex optimization problems [3] and are widely applied across many engineering and scientific fields.

In recent years, researchers have proposed numerous metaheuristic algorithms, including Newton-Raphson-Based Optimizer (NRBO) [4], Artemisinin Optimization (AO) [5], Ivy Optimization Algorithm (IVY) [6], Tianji’s Horse Racing Optimization (THRO) [7], Projection-Iterative-Methods-based Optimizer (PIMO) [8], Holistic Swarm Optimization (HSO) [9], Artificial Lemming Algorithm (ALA) [10], Genetic Algorithm (GA) [11], Chaos Game Optimization (CGO) [12], Gradient-Based Optimizer (GBO) [13], Slime Mold Algorithm (SMA) [14], Marine Predators Algorithm (MPA) [15], Polar Lights Optimization (PLO) [16], Parrot Optimizer (PO) [17], Black-winged Kite Algorithm (BKA) [18], GOOSE algorithm (GOOSE) [19], Hippopotamus Optimization Algorithm (HO) [20], PID-based Search Algorithm (PIA) [21], Fully Informed Search Algorithm (FISA) [22], Sinh Cosh Optimizer (SCHO) [23], An Enhanced Snake Optimizer Algorithm (ESOA) [24], Tyrannosaurus Optimization Algorithm (TOA) [25], and many others. However, to date, no universally applicable optimization algorithm capable of effectively solving all engineering and numerical optimization problems has been developed [26]. Therefore, researchers are committed to improving existing metaheuristic algorithms by integrating specific strategies to develop various variants, aiming to enhance their performance, robustness, and adaptability to specific problem categories. These improvement strategies have significantly expanded the range of metaheuristic algorithms. For example, Qiao et al. [27] proposed an Improved Red-Billed Blue Magpie Optimization (IRBMO) algorithm by integrating multiple strategies such as Logistic-Tent chaotic initialization, a dynamic balance factor, and dual-mode perturbation. This effectively enhanced population diversity, balanced search capability, and addressed the issues of premature convergence and performance degradation of the original algorithm in high-dimensional optimization. Lv et al. [28] proposed a Multi-Strategy Improved Dung Beetle Optimizer (MIDBO) by incorporating strategies such as improved chaotic initialization, a nonlinear dynamic balance factor, and multi-population differential co-evolution. This effectively enhanced population diversity, balanced exploration and exploitation capabilities, and resolved the tendencies of premature convergence and insufficient accuracy of the original algorithm in complex optimization problems. Chen et al. [29] proposed the Butterfly Search and Triangular Walk Crested Porcupine Optimizer (BTCPO), which effectively addressed the shortcomings of the original Crested Porcupine Optimizer in terms of convergence speed and local exploitation accuracy, achieving a dynamic balance between exploration and exploitation. It demonstrated significant advantages on both benchmark functions and engineering design problems.

The Superb Fairy-wren Optimization Algorithm (SFOA) is a metaheuristic inspired by the social behavior of the superb fairy-wren [30]. While the Superb Fairywren Optimization Algorithm (SFOA) is inspired by the complex social behaviors of the superb fairywren, which establish a multi-phase search strategy framework and demonstrate considerable potential for solving complex problems, the original algorithm is constrained by several structural limitations. For example, its reliance on fixed weighting factors hinders an adaptive balance between exploration and exploitation; its simplistic and random population initialization method often results in insufficient diversity; and its escape mechanism proves inefficient when addressing complex multimodal problems. These shortcomings collectively limit the algorithm’s performance, increasing its susceptibility to local optima and reducing solution accuracy. Therefore, this study selects the SFOA as a basis for enhancement, aiming to mitigate these structural weaknesses through targeted modifications, thereby unlocking its latent potential and developing a more robust and effective optimization approach. To address these limitations, this paper proposes an Improved Superb Fairy-wren Optimization Algorithm (ISFOA), which integrates four strategies—Chebyshev chaotic mapping, an adaptive weighting factor, Cauchy–Gaussian mutation, and t-distribution perturbation—to better balance global exploration and local exploitation. An ablation study using the CEC 2021 benchmark was conducted to assess the contribution of each strategy. The effectiveness of ISFOA was further evaluated through comprehensive comparisons with eight other metaheuristic algorithms on the CEC 2005 and CEC 2021 benchmark suites. Additionally, the practical performance of ISFOA was examined by applying it to seven engineering design problems and comparing the results with those obtained by the eight other optimizers. The main contributions of this paper are as follows:

(1) The Improved Superb Fairy-wren Optimization Algorithm (ISFOA) is proposed, which integrates four strategies: Chebyshev chaotic mapping, an adaptive weighting factor, Cauchy–Gaussian mutation, and t-distribution perturbation.

(2) The Chebyshev chaotic mapping strategy is introduced to enhance the traversal and uniformity of the initial population distribution in the solution space.

(3) An adaptive weighting factor is designed to balance exploration and exploitation throughout the entire iterative process, a challenge in the original SFOA.

(4) The Cauchy–Gaussian mutation strategy is incorporated to improve population diversity.

(5) The t-distribution perturbation strategy is further introduced to enhance the robustness and solution quality of ISFOA, as well as its ability to balance global exploration and local exploitation.

(6) Ablation and comparative experiments are conducted between ISFOA and several classical and newly proposed metaheuristic algorithms on different benchmark sets. Results demonstrate that ISFOA exhibits significant advantages in robustness, convergence accuracy, and other performance metrics.

(7) The effectiveness of ISFOA is also validated on real-world engineering problems.

The structure of this paper is as follows: Section 1 presents the introduction; Section 2 describes the Superb Fairy-wren Optimization Algorithm (SFOA); Section 3 details the improvement strategies applied to SFOA; Section 4 presents ablation and comparative experiments between ISFOA and other metaheuristic algorithms on different benchmark sets; Section 5 validates the performance of ISFOA on practical engineering problems; finally, conclusions and discussions are provided in Section 6.

## 2. Superb Fairy-Wren Optimization Algorithm

The Superb Fairy-wren Optimization Algorithm is a meta-heuristic algorithm that emulates the natural behaviors of the superb fairy-wren—including foraging, breeding, chick-rearing, and predator evasion—to search for optimal solutions to complex problems.

### 2.1. Population Initialization

Similarly to other meta-heuristic optimization algorithms, SFOA employs random initialization for population initialization:

(1) $$X_{i,j}=\left(ub-lb\right)\times rand+lb$$

where $X_{i,j}$ represents the position of the *i*-th individual in the *j*-th dimension; ub denotes the upper bound of the search space; lb denotes the lower bound of the search space; and rand denotes random numbers within the range.

### 2.2. Young Bird Growth Stage

During the Young Bird Growth Stage, the position of each individual in the population is updated by simulating the growth dynamics of nestlings. SFOA models the process of individual position updates in the solution space: individuals continuously learn from extensive experience and rapidly adjust their positions throughout this growth stage.

(2) $$X_{i,j}^{t+1}=X_{i,j}^{t}+(lb+(ub-lb)\times rand),\;r>0.5$$

where $X_{i,j}^{t+1}$ denotes the position of the *i*-th individual in the *j*-th dimension at time step *t* + 1; $X_{i,j}^{t}$ denotes the position of the *i*-th individual in the *j*-th dimension at time step *t*; r denotes the proportional coefficient for juveniles.

### 2.3. Breeding and Feeding Stage

When environmental risk factors are low and the population is dominated by adult birds, the algorithm transitions into the Breeding and Feeding Stage. In SFOA, to assess the level of environmental danger, the environmental risk value is first calculated using Equation (4) to simulate risk fluctuations. When the computed risk value s is sufficiently small, it indicates a relatively safe environment, and the algorithm proceeds to the Breeding and Feeding Stage.

(3) $$X_{i,j}^{t+1}=X_{b}\times C+\left(X_{b}-X_{i,j}^{t}\right)\times p,\;r<0.5\;\mathrm{and}\;s<20$$

(4) $$s=r_{1}\times 20+r_{2}\times 20$$

(5) $$p=\sin((ub-lb)\times 2+(ub-lb)\times \left(\frac{FES}{MaxFES}\right)\times 2)$$

where $X_{b}$ denotes the position of the current optimal individual; C denotes the weighting factor, which is set to 0.8 in the original algorithm; s denotes the environmental danger factor; p denotes the process of the gradual expansion of the activity range during the rotational tutoring among birds; FEs denotes the current number of function evaluations; MaxFEs denotes the maximum number of function evaluations.

### 2.4. Avoiding Natural Enemies Stage

In the Avoiding Natural Enemies Stage, the SFOA updates individual positions by simulating the defensive responses of prey to predation. When a simulated individual is detected, it engages in rapid displacement and high-frequency perturbations to evade tracking. Concurrently, it emits warning signals that trigger adaptive responses from other members of the population. Collectively, these modeled behaviors form the foundation for the position-update mechanism in this stage.

(6) $$X_{i,j}^{t+1}=X_{b}+X_{i,j}^{t}\times l\times k,\;r<0.5\;\mathrm{and}\;s>20$$

(7) $$k=0.2\times \sin\left(\frac{\pi }{2}-\frac{\pi }{2}\times \frac{FEs}{MaxFEs}\right)$$

where l denotes the random step size generated by Lévy flight; k denotes the escape distance coordination factor.

## 3. Improved Superb Fairy-Wren Optimization Algorithm

### 3.1. Chebyshev Chaotic Map

To address the problem that the population initialization method in the standard SFOA tends to produce uneven population distribution and inadequate exploration of the solution space, this study employs a Chebyshev chaotic map to generate the initial population. This approach enhances the ergodicity and distribution uniformity of the population across the solution space, thereby improving the algorithm’s global exploration capability in the early search stages and increasing its computational efficiency. The working principle of the Chebyshev chaotic mapping strategy is described as follows:

(8) $$X(t+1)\;=\;\cos\left[\varphi \cdot \arccos X\left(t\right)\right]\;$$

where φ denotes the initialization coefficient; $X\left(t\right)\in \left[-1,1\right]$ denotes the function value of the *t*-th iteration; $X\left(t+1\right)$ denotes the function value of the (*t* + 1)-th iteration.

### 3.2. Adaptive Weighting Factor

In the breeding and brooding phase of the SFOA, the weighting coefficient *C* is a key parameter that controls the degree to which individuals learn from the optimal one. In the original SFOA, *C* is fixed at 0.8, which results in inflexible search behavior and disrupts the balance between exploration and exploitation. To overcome this limitation, the ISFOA replaces it with an adaptive weight *C(t)*, bounded within [0.1, 1]. Setting $C_{\max}=1$ allows the algorithm to closely follow the global optimum in early iterations, facilitating broad exploration; setting $C_{\min}=0.1$ ensures that, in later stages, the search shifts toward local refinement while preserving a baseline of global guidance. The weight decays nonlinearly from 1 to 0.1, driving a smooth adaptive search process. This modification not only better simulates biological behavior, but also meets the optimization-theoretic requirement for dynamic equilibrium between exploration and exploitation.

To verify the sensitivity of the adaptive weighting factor, we selected five representative functions (F2, F3, F6, F9, F10) from the CEC 2021 benchmark and tested 16 parameter combinations formed by varying $C_{\min}$ ∈ {0.05, 0.1, 0.15, 0.2} and $C_{\max}$ ∈ {0.8, 0.9, 1.0, 1.1}.

**Figure 1:**
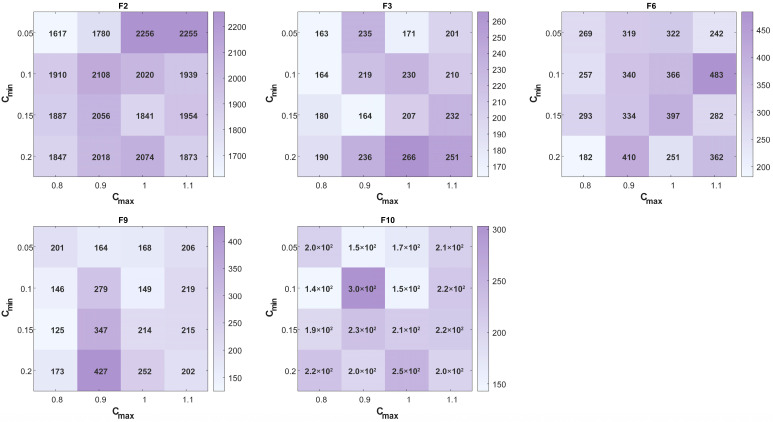
Heat map of adaptive weighting factor.

The results, visualized in a series of heatmaps (Figure 1), show that the ISFOA maintains stable and high performance across diverse optimization problems within the tested parameter range. Specifically, a contiguous region of darker shading—indicating better performance—surrounds the chosen parameter set (0.1, 1.0). This pattern suggests that performance remains robust under slight parameter variations, demonstrating low sensitivity around this configuration.

The adaptive weighting factor operates on the following principle:

(9) $$C(t)=C_{\min}+\left(C_{\max}-C_{\min}\right)\cdot \left[1-{\left(\frac{t}{T_{\max}}\right)}^{0.5}\right]$$

where $C_{\min}$ denotes the minimum value of the weight, which is set to 0.1 in this study; $C_{\max}$ denotes the maximum value of the weight, which is set to 1 in this study; and $T_{\max}$ denotes the maximum number of time steps.

### 3.3. Cauchy–Gaussian Mutation

Population diversity is a critical factor influencing the performance of optimization algorithms. During the search process, its decline often leads to premature convergence to local optima. To address this, the paper introduces a Cauchy–Gaussian hybrid mutation strategy to perturb individuals. This approach combines the heavy-tailed properties of the Cauchy distribution, which facilitate escaping local optima, with the local search properties of the Gaussian distribution, thereby effectively maintaining population diversity and promoting global convergence. The principle of the Cauchy–Gaussian mutation strategy is described as follows:

(10) $$U_{b}=X_{b}\cdot \left[1+\omega_{1}\cdot C_{a}\left(0,\sigma^{2}\right)+\omega_{2}\cdot G_{a}\left(0,\sigma^{2}\right)\right]$$

(11) $$\sigma =\left\{\begin{array}{cc} 1 & f\left(X_{b}\right)<f\left(X_{i,j}\right)\\ \exp\left(\frac{f\left(X_{b}\right)-f\left(X_{i,j}\right)}{\left|f\left(X_{b}\right)\right|}\right) & f\left(X_{b}\right)\geq f\left(X_{i,j}\right) \end{array}\right.$$

where $U_{b}$ denotes the Cauchy–Gaussian mutated individual; $\omega_{1}=1-t^{2}/T_{\max}^{2}$; $\omega_{2}=t^{2}/T_{\max}^{2}$; $\sigma^{2}$ denotes the standard deviation; $C_{a}\left(0,\sigma^{2}\right)$ denotes a random variable following the Cauchy distribution; $G_{a}\left(0,\sigma^{2}\right)$ denotes a random variable following the Gaussian distribution.

### 3.4. T-Distribution Perturbation

To enhance the robustness, solution quality, and exploration–exploitation balance of the SFOA, a t-distribution perturbation strategy is introduced following the individual position update. This strategy integrates the strengths of both Gaussian and Cauchy mutations, promoting a dynamic search trade-off and alleviating the tendency to converge prematurely or stagnate in local optima during later iterations. Specifically, the iteration number serves as the degrees of freedom of the t-distribution, moderating the magnitude of perturbation. In early iterations, the perturbation resembles a Cauchy distribution, promoting global exploration; in later stages, it approximates a Gaussian distribution, favoring local refinement. This design effectively prevents premature convergence and enhances the overall search efficiency of the algorithm.

The t-distribution perturbation strategy operates as follows:

(12) $$X_{i,j}(t+1)=X_{i,j}(t)+X_{i,j}(t)\times t(F_{iter})$$

where $t(F_{iter})$ denotes the t-distribution with degrees of freedom being the number of iterations.

This study proposes a multi-strategy enhancement to the Superb Fairywren Optimization Algorithm (SFOA) designed to address specific limitations at distinct stages of the search process. The core improvement lies not in a simple combination of four strategies, but in a temporally structured and functionally complementary framework that fosters synergistic interaction. The framework systematically integrates: (1) a Chebyshev chaotic map for generating a uniformly distributed initial population, thereby establishing a robust foundation for global exploration; (2) an adaptive weight factor to dynamically regulate the trade-off between exploration and exploitation throughout the iterative process; (3) a Cauchy–Gaussian hybrid mutation introduced in the mid-phase, leveraging the global exploration capability of the Cauchy operator and the local refinement strength of the Gaussian operator to sustain population diversity and counteract premature convergence; and (4) a t-distribution perturbation operator applied in later iterations, which facilitates a smooth transition between the local-focused Gaussian pattern and the exploration-oriented Cauchy pattern, thereby enhancing the quality and robustness of the final convergence. The effectiveness of this coordinated, phased strategy combination is demonstrated conclusively through systematic ablation experiments. The performance of the complete ISFOA significantly exceeds that of any variant lacking a single component, providing strong evidence for the theoretical rationale and practical utility of the proposed approach in simultaneously improving convergence accuracy, speed, and stability.

### 3.5. The Flowchart for ISFOA

**Figure 2:**
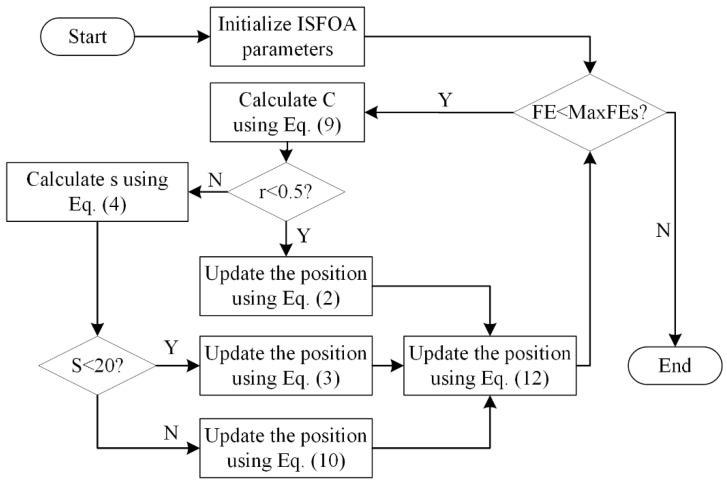
The flowchart of the Improved Superb Fairy-wren Optimization Algorithm.

To show the specific steps of ISFOA more clearly, its pseudo-code and flowchart are shown here, as shown in Algorithm 1 and Figure 2.
**Algorithm 1** Pseudocode of ISFOA1. Input: The number of candidate solution N, Dimension D, The max number of fitness evaluation MaxFEs2. Output: The best candidate solution $X_{best}$3. Generate the initial population using Equation (8)4. **While** FEs < MaxFEs5.   Calculate C using Equation (9)6.   **For** i = 1:N7.    **If** r > 0.58.     Update the position using Equation (2)9.    **Else**10.     Calculate s using Equation (4)11.     **If** s < 2012.      Update the position using Equation (3)13.     **Else**14.      Update the position using Equation (10)15.     **End if**16.    **End if**17.   **End for**18.   Evaluate fitness of each candidate solution19.   FEs = FEs + N20. **End while**

### 3.6. The Time Complexity of ISFOA

For an optimization problem with population size N and dimension D, given a maximum iteration count T, the overall time complexity of the original SFOA is O(N × D × T). ISFOA incorporates four enhancement strategies. First, the Chebyshev Chaotic Map replaces random initialization, requiring O(N × D) operations once, thus contributing negligibly to per-iteration complexity. Second, the Adaptive Weighting Strategy dynamically updates the weight C during breeding and brooding, adding about 4 × N × Doperations per iteration. Third, the Cauchy–Gaussian Mutation perturbs the best individual, introducing roughly 5 × D operations per iteration. Finally, the t-distribution Perturbation acts on the whole population after position updates, adding about 2 × N × D operations per iteration.

Overall, the time complexity of ISFOA remains O(N × D × T). Although the added strategies increase per-iteration computation, they incur only constant-factor overhead and do not change the asymptotic order. Importantly, the resulting gains in convergence speed and solution quality outweigh this overhead. Compared to SFOA and other benchmarks, ISFOA typically reaches better solutions in fewer iterations, achieving a favorable trade-off between computational cost and performance. Thus, ISFOA not only attains superior optimization results but also exhibits higher overall efficiency on complex problems.

## 4. Performance Evaluation of the Improved Superb Fairy-Wren Optimization Algorithm

All experiments in this section were conducted on a conventional personal computer running MATLAB R2023a, configured with a Windows 11 (64-bit) OS, an Intel(R) Core(TM) i7-6700HQ CPU, and 24 GB of RAM. The parameters of the comparison algorithm are shown in Table 1.

### 4.1. Test Function Configuration

To evaluate the performance of the ISFOA, ablation studies and comparative experiments were conducted using benchmark functions from the CEC2021, CEC2005 [31], and CEC2021 [32] test suites, as detailed in Table 2 and Table 3. The CEC2005 benchmark comprises 23 functions, categorized into unimodal (F1–F5), multimodal (F6–F13), and fixed-dimensional multimodal (F14–F23) types, which collectively assess convergence accuracy, speed, and ability to avoid local optima. The CEC2021 benchmark includes 10 functions, divided into basic (F1–F6), hybrid (F7–F8), and composite (F9–F10) categories, providing further evaluation of the algorithm’s robustness and global search capability in complex solution spaces.

### 4.2. Abolition Experiment

The experimental settings in the ablation study are consistent with those in the comparative experiments: the population size is set to N = 30, the maximum number of iterations is 1000, and each algorithm is independently run 30 times to obtain statistical results.

To assess the individual contributions of the four strategies to the overall performance of SFOA (Table 4), an ablation study was conducted using the CEC 2021 benchmark functions. The ablation variants are defined as follows: ISFOA1 omits the Chebyshev chaotic map, reverting to random initialization; ISFOA2 removes the adaptive weighting factor, employing a fixed weight (C = 0.8); ISFOA3 excludes the Cauchy–Gaussian mutation strategy; and ISFOA4 omits the t-distribution perturbation strategy. The results (Table 5) evaluate the independent effect of each core strategy in the Improved SFOA. The findings demonstrate the following: the t-distribution perturbation most significantly enhances the algorithm’s ability to explore complex search spaces; the adaptive weighting factor plays a key role in multimodal optimization; the Cauchy–Gaussian mutation effectively maintains population diversity; and the Chebyshev chaotic map facilitates a more uniform initial population distribution. The complete ISFOA outperforms all ablation variants in convergence accuracy, stability, and robustness, confirming the effectiveness of the multi-strategy cooperative design.

Furthermore, we conducted the Wilcoxon rank-sum test on ISFOA and its variants, as shown in Table 6. The Wilcoxon rank-sum test is a nonparametric statistical test used to determine whether a significant difference exists between two independent samples. In the experiments, the significance level was set at 0.05. A significance level exceeding 0.05 indicates no statistically significant difference, while a value below 0.05 denotes a significant performance difference. In this table, the symbols “+”, “−”, and “=” represent cases where ISFOA performs significantly better, significantly worse, or shows no significant difference, respectively.

The results indicate that all four enhancement strategies integrated into the ISFOA positively impact the performance of the original SFOA, though their scope and degree of significance vary. Specifically, the Chebyshev chaotic mapping strategy performs comparably to the original random initialization on most test functions, yet it demonstrates distinct advantages on the composite function F10, providing a better initial population distribution for handling complex problems. The adaptive weighting factor shows widespread and consistent performance gains, leading to significant improvements across all test functions. The Cauchy–Gaussian mutation strategy provides clear and targeted enhancements for optimizing hybrid and composite functions. The t-distribution perturbation strategy yields significant performance improvements on all functions.

### 4.3. Comparative Experimental Study on the CEC 2005

To evaluate the optimization performance of the ISFOA, comparative experiments were conducted using the CEC 2005 benchmark test suite. The algorithms selected for comparison include SFOA, Particle Swarm Optimization(PSO) [33], Gray Wolf Optimizer (GWO) [34], Sparrow Search Algorithm (SSA) [35], Rime Optimization Algorithm (RIME) [36], Coati Optimization Algorithm (COA) [37], Subtraction-Average-Based Optimizer (SABO) [38], and Dung Beetle Optimizer (DBO) [39], representing eight established metaheuristic optimizers. Comprehensive results demonstrate that the multi-strategy Improved SFOA (ISFOA) significantly outperforms the original SFOA and other mainstream algorithms in solution accuracy, convergence speed, and robustness on this benchmark.

The numerical results for the CEC 2005 functions are presented in Table 7. A synthesis of outcomes from both the CEC 2005 and CEC 2021 suites allows a performance analysis along two core dimensions: solution accuracy and robustness. In terms of solution accuracy, ISFOA shows a marked advantage on the vast majority of test functions. After 30 independent runs, its best, average, and median values consistently reach or closely approach the theoretical global optimum across unimodal, multimodal, and fixed-dimensional functions. Particularly on high-dimensional or highly multimodal complex functions, ISFOA reliably finds solutions of superior quality, with errors often orders of magnitude lower than those of other algorithms. Regarding algorithm robustness, ISFOA exhibits strong stability. The standard deviation of its results is generally very low, reaching zero for some functions, which indicates minimal sensitivity to the randomness of initialization and reliable convergence in every trial. In contrast, the performance of the other compared algorithms fluctuates more considerably. Their best and average values deviate significantly from the theoretical optimum, accompanied by higher standard deviations, reflecting less stable performance and lower success rates. The high accuracy and robustness of ISFOA validate that the proposed multi-strategy enhancements effectively improve its overall capability to handle diverse search landscapes.

**Figure 3:**
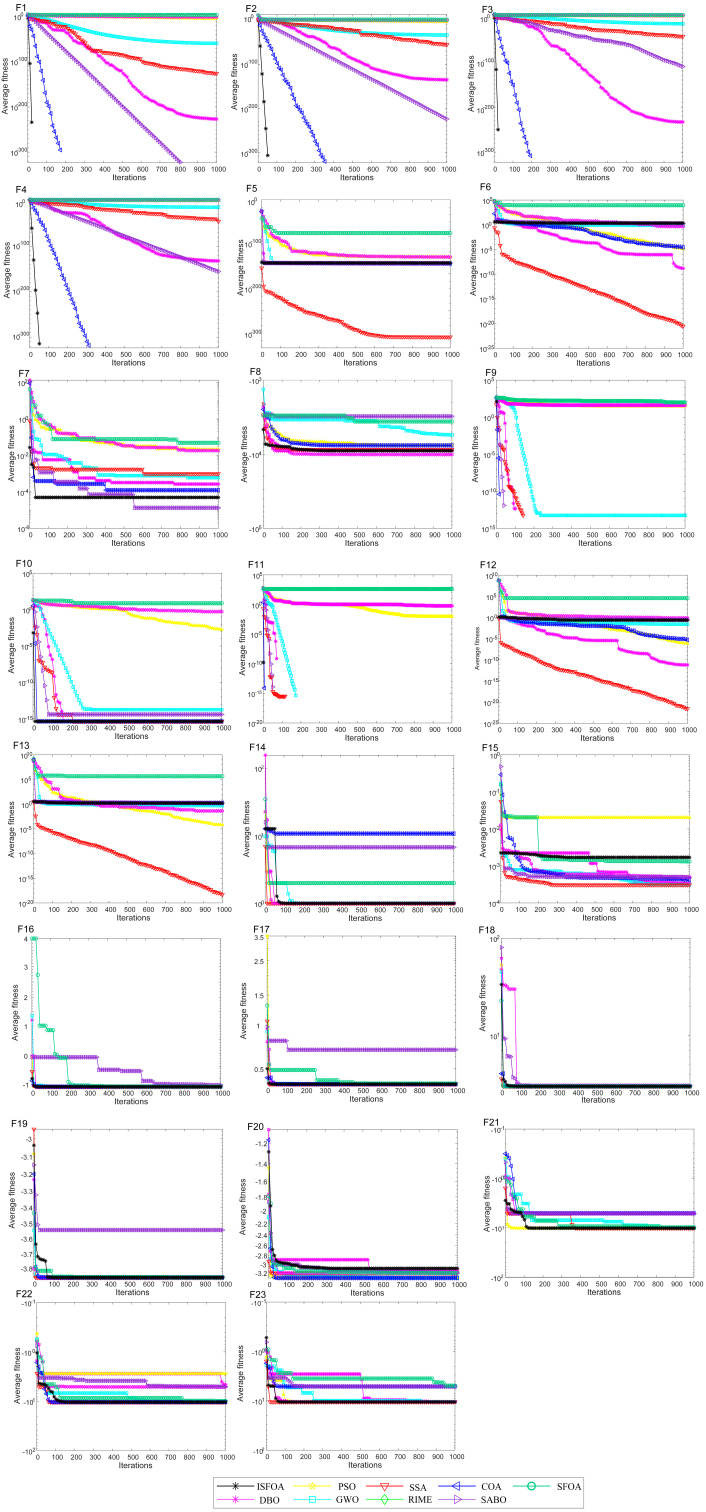
CEC2005 convergence iteration curve.

The convergence curves on the CEC 2005 test set are shown in Figure 3. Compared to SFOA, PSO, GWO, SSA, RIME, COA, SABO, and DBO, ISFOA demonstrates superior convergence behavior on most functions. On unimodal functions, its convergence curve descends steeply from the early iterations and stabilizes rapidly near the theoretical optimum, verifying the efficiency of its improved initialization and adaptive mechanisms in simpler search spaces. On multimodal functions, ISFOA’s curve decreases steadily in a “step-wise” manner, effectively escaping local optima. In contrast, algorithms like SFOA and PSO often stagnate prematurely. On fixed-dimensional multimodal functions, ISFOA also converges quickly and stably, with a smooth curve and final accuracy markedly better than that of other algorithms, some of which exhibit slow convergence, oscillatory behavior, or premature stagnation.

**Figure 4:**
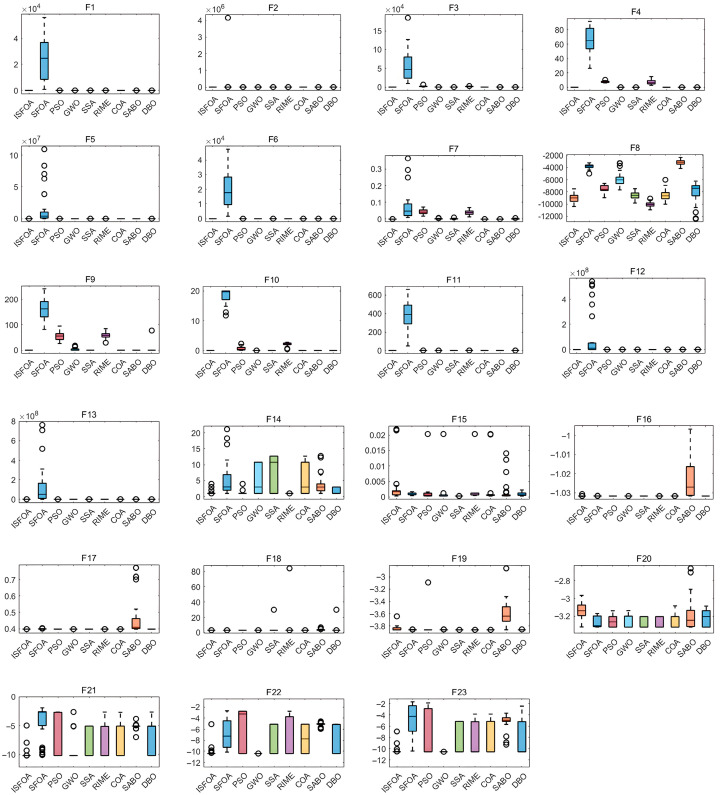
CEC2005 box-and-line plot.

The box plots for the CEC 2005 test set are shown in Figure 4, visually summarizing the stability and result quality distribution for each algorithm over 30 independent runs. ISFOA’s box plots show a clear advantage on most functions. For functions where the theoretical optimum is attainable, ISFOA’s box collapses to a single line at the optimum with no outliers, indicating 100% accurate convergence and absolute stability. For more challenging functions, its box is typically the shortest and positioned closest to the optimum. In contrast, the box plots for the original SFOA and other algorithms commonly display elongated boxes, medians distant from the optimum, and numerous outliers, reflecting high result variability, unstable performance, and sensitivity to initial conditions or random factors.

Table 8 presents the results of the Wilcoxon rank-sum test (significance level α = 0.05), where the symbols “+”, “−”, and “=” denote that ISFOA performs significantly better, significantly worse, or not significantly different, compared to each algorithm, respectively.

The results show that ISFOA achieves a statistically significant advantage over PSO, GWO, SSA, and RIME on all 23 test functions. Against SABO and DBO, ISFOA also demonstrates strong competitiveness, securing a significant advantage in 22 out of 23 comparisons. When compared with COA, ISFOA performs significantly better on 16 functions, with no significant difference observed on the remaining 7.

In summary, across all benchmark comparisons, the number of cases in which ISFOA holds a significant advantage far exceeds those with no significant difference. This statistically confirms that ISFOA achieves an overall performance improvement in terms of solution accuracy, robustness, and convergence, demonstrating clear comprehensive advantages over the state-of-the-art algorithms included in this study.

### 4.4. Comparative Experimental Study on the CEC 2021

The results for the CEC 2021 test functions are presented in Table 9. ISFOA demonstrates significant advantages in solution accuracy, stability, and robustness.

On the basic functions (F1–F6), ISFOA consistently attains the theoretical optimum of 0, indicating precise convergence to the global optimum in all 30 independent runs. In contrast, algorithms such as SFOA and PSO produce results that are several orders of magnitude worse, highlighting ISFOA’s superior accuracy and reliability on these functions. On the hybrid functions (F7–F8), ISFOA maintains an extremely high convergence success rate, with both its best and mean values at zero and a near-zero standard deviation, demonstrating its efficacy on complex, multi-modal landscapes. On the composite functions (F9–F10), ISFOA remains stable and consistently locates the theoretical optimum, whereas other algorithms exhibit performance degradation, with some failing to approach the optimal solution effectively. This further confirms ISFOA’s strong global exploration capability and its ability to escape local optima.

**Figure 5:**
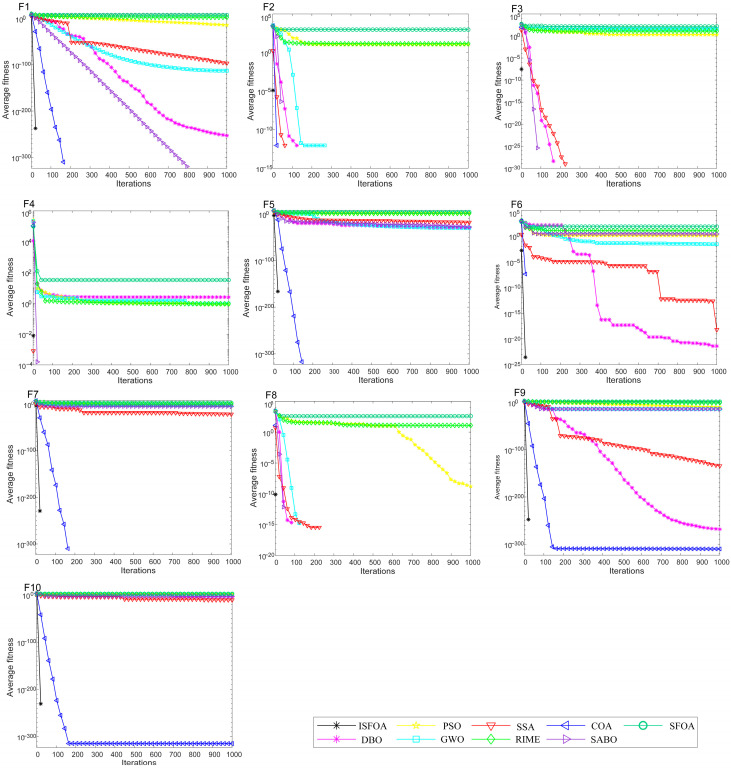
CEC2021 convergence iteration curve.

The convergence curves for CEC 2021 are shown in Figure 5. They visually illustrate ISFOA’s significant advantage, with convergence behavior adapting to function complexity. On basic functions, ISFOA’s curve descends steeply and stabilizes rapidly at the optimum without oscillation. On hybrid functions, the curves show excellent adaptability across search regions, converging smoothly without the plateaus or fluctuations seen in other algorithms. On the most challenging composite functions, ISFOA exhibits a steady, step-wise decline, consistently escaping local optima. In contrast, comparison algorithms such as SFOA and PSO often stagnate prematurely. This demonstrates the effectiveness of ISFOA in maintaining population diversity and ensuring global convergence.

**Figure 6:**
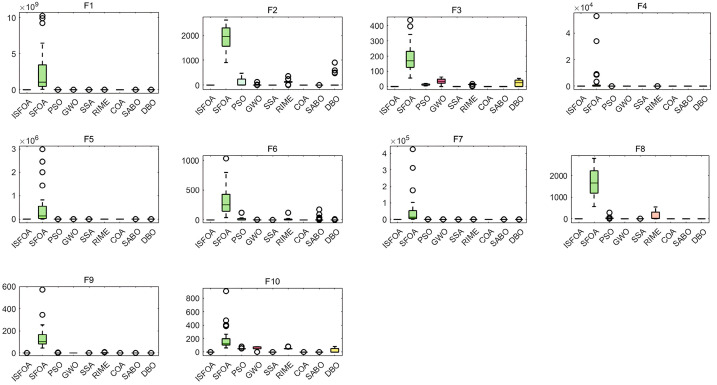
CEC2021 box-and-line plot.

The box plots for CEC 2021 are shown in Figure 6, further confirming ISFOA’s exceptional stability and robustness. Across all ten functions, ISFOA’s box plots show consistent superiority. On functions where the theoretical optimum is attainable, the box collapses to a single line at the optimum with no outliers. Even on composite functions where other algorithms struggle, ISFOA’s box is the shortest, and its median is closest to the optimum, indicating highly concentrated, high-quality solutions. In contrast, other algorithms typically exhibit elongated boxes, medians far from the optimum, widely dispersed edges, and numerous outliers. This reflects substantial result fluctuation, unstable performance, and high sensitivity to initial conditions.

The results of the Wilcoxon rank-sum test are presented in Table 10. In the experiments, the significance level was set at 0.05. A significance level exceeding 0.05 indicates no statistically significant difference, while a value below 0.05 denotes a significant performance difference. In this table, the symbols “+”, “−”, and “=” denote that ISFOA performs significantly better, significantly worse, or shows no significant difference, respectively.

Specifically, compared to the original SFOA, ISFOA demonstrates statistically significant superiority across all 10 test functions, fully validating the effectiveness of the improvement strategies proposed in this paper. In comparison with traditional algorithms (e.g., PSO and RIME), ISFOA also exhibits comprehensive superiority, performing significantly better on all test functions, which highlights its strong competitiveness in solving optimization problems. Compared to GWO and DBO, ISFOA shows significant advantages on 9 functions, with comparable performance only on function F8. This is because the specific structure of F8 enables both ISFOA and GWO to converge to the global optimum. Against SSA and SABO, ISFOA performs significantly better on six and seven functions, respectively, while no significant difference is observed on the remaining functions. Compared to COA, ISFOA demonstrates better performance on functions F9 and F10, indicating its greater competitiveness in solving hybrid and composition functions.

In summary, the experimental results based on the CEC2021 benchmark set indicate that the overall performance of ISFOA is superior to all compared algorithms, including the original SFOA, PSO, GWO, SSA, RIME, COA, SABO, and DBO.

## 5. Engineering Applications of ISFOA

To assess the performance of ISFOA on practical engineering constrained optimization problems, seven typical problems were selected and solved using ISFOA and various other metaheuristic algorithms, as listed in Table 11. Engineering constrained optimization is generally challenging due to the frequent presence of multiple highly nonlinear and complex constraints. In this study, the penalty function method is employed to handle these constraints by assigning a sufficiently large penalty to infeasible solutions, thereby converting the original constrained problem into an unconstrained formulation. Table 12 presents the best value, mean value, standard deviation, and corresponding ranking obtained from 30 independent runs of each algorithm.

The results in Table 12 show that ISFOA is highly effective in solving the selected engineering optimization problems. It outperforms all other compared algorithms on six of the seven problems, with its result on problem P5 being only slightly less competitive than that of DBO. Overall, ISFOA successfully handles constrained optimization in practical engineering contexts, demonstrating excellent performance, and its comprehensive results are clearly superior to those of the other algorithms evaluated. 

## 6. Conclusions

This study proposes an Improved Superb Fairy-wren Optimization Algorithm. The algorithm enhances the original Superb Fairy-wren Optimization Algorithm by integrating four strategies: a Chebyshev Chaotic Map, an adaptive weighting factor, Cauchy–Gaussian mutation, and t-distribution perturbation. These improvements systematically bolster the algorithm’s capacity to balance global exploration and local exploitation, maintain population diversity, and achieve higher convergence accuracy.

Ablation studies confirm the critical role of each strategy. The Chebyshev Chaotic Map ensures a uniform initial population distribution. The adaptive weighting factor dynamically balances exploration and exploitation during multimodal optimization. Cauchy–Gaussian mutation effectively preserves population diversity, while t-distribution perturbation provides a robust mechanism to escape local optima in later iterations, which is particularly impactful in complex search spaces.

Experimental evaluations on the CEC2005 and CEC2021 benchmark suites show that ISFOA significantly outperforms several mainstream metaheuristic algorithms—including DBO, COA, GWO, PSO, SSA, RIME, and SABO—in terms of the mean, best, and standard deviation of results. This demonstrates ISFOA’s superior solution accuracy, robustness, and convergence speed, as further validated by the Wilcoxon rank-sum test. Moreover, when applied to seven real-world constrained engineering problems (e.g., welded beam design, tension/compression spring design, and pressure vessel design), ISFOA also exhibits excellent performance in both solution quality and stability, underscoring its practical utility.

Despite its strong overall performance, ISFOA has limitations. Its convergence precision on certain test functions can be improved, and the incorporation of multiple strategies increases computational complexity. Future work will therefore focus on the following: (1) optimizing the adaptive parameter adjustment mechanism; (2) developing hybrid models by combining ISFOA with other intelligent algorithms; (3) extending its application to high-dimensional, multi-objective, and large-scale optimization problems, as well as to dynamic systems such as fault diagnosis and remaining useful life prediction; and (4) validating its competitiveness in more complex engineering scenarios, including UAV path planning, feature selection, and hyperparameter optimization. These efforts aim to advance the practical application of metaheuristic algorithms in broader domains.

## Figures and Tables

**Table 1:** Parameters of the comparison algorithm.

Algorithm	Parameters Setting
SFOA	C = 0.8, T = 0.5, β = 1.5
PSO	c1 = 1.5, c2 = 1.5
GWO	a = 2 → 0
SSA	P = 0.2, N = 20
RIME	W = 5
COA	C = 2 → 0, temp ∈ [20, 35]
SABO	-
DBO	P = 0.2

**Table 2:** Test Function for CEC2005.

Type	ID	Name
Unimodal functions	F1	Sphere Function
	F2	Schwefel’s Problem 2.22
	F3	Schwefel’s Problem 1.2
	F4	Schwefel’s Problem 2.21
	F5	Generalized Rosenbrock’s Function
	F6	Step Function
	F7	Quartic Function with Noise
Multimodal functions	F8	Generalized Schwefel’s Problem 2.26
	F9	Generalized Rastrigin’s Function
	F10	Ackley’s Function
	F11	Generalized Griewank’s Function
	F12	Generalized Penalized Function 1
	F13	Generalized Penalized Function 2
Fixed-dimension Multimodal	F14	Shekel’s Foxholes Function
	F15	Kowalik’s Function
	F16	Six-Hump Camel-Back Function
	F17	Branin Function
	F18	Goldstein-Price Function
	F19	Hartman’s Family (3, 4)
	F20	Hartman’s Family (6, 4)
	F21	Shekel’s Family (5, 4)
	F22	Shekel’s Family (7, 4)
	F23	Shekel’s Family (10, 4)

**Table 3:** Test Function for CEC2021.

Type	ID	Name
Basic Function	F1	Shifted and Full Rotated Zakharov Function
	F2	Shifted and Full Rotated Rosenbrock’s Function
	F3	Shifted and Full Rotated Expanded Schaffer’s f6 Function
	F4	Shifted and Full Rotated Non-Continuous Rastrigin’s Function
	F5	Shifted and Full Rotated Lunacek bi-Rastrigin Function
	F6	Shifted and Full Rotated Expanded Rosenbrock’s plus Griewangk’s Function
Hybrid Function	F7	Hybrid Function 1
	F8	Hybrid Function 2
Composition Function	F9	Composition Function 1
	F10	Composition Function 2

**Table 4:** Various SFOA variants with different strategies.

ID	ISFOA	SFOA	ISFOA1	ISFOA2	ISFOA3	ISFOA4
Chebyshev Chaotic Map	Y	N	N	Y	Y	Y
Adaptive Weighting Factor	Y	N	Y	N	Y	Y
Cauchy–Gaussian Mutation	Y	N	Y	Y	N	Y
T-distribution Perturbation	Y	N	Y	Y	Y	N

**Table 5:** The result of the abolition experiment.

ID	Index	ISFOA	SFOA	ISFOA1	ISFOA2	ISFOA3	ISFOA4
F1	min	0	4.19 × 10^7^	0	207.62	0	4 × 10^−14^
	std	0	2.9 × 10^9^	0	4.25 × 10^5^	0	2.03 × 10^−1^
	avg	0	2.27 × 10^9^	0	1.86 × 10^5^	0	3.72 × 10^−2^
	median	0	1.21 × 10^9^	0	4.4 × 10^4^	0	6 × 10^−7^
	worse	0	1.36 × 10^10^	0	1.97 × 10^6^	0	1.11
F2	min	0	951.35	0	345.24	0	1.36 × 10^−11^
	std	0	454.26	0	253.75	0	455.11
	avg	0	1.88 × 10^3^	0	713.06	0	483.91
	median	0	1.85 × 10^3^	0	702.88	0	460.57
	worse	0	2.97 × 10^3^	0	1.42 × 10^3^	0	1.44 × 10^3^
F3	min	0	81.35	0	12.52	0	1.78 × 10^−14^
	std	0	52.67	0	17.14	0	18
	avg	0	158.33	0	47.25	0	24.64
	median	0	148.96	0	40.51	0	29.09
	worse	0	296.27	0	82.45	0	56.1
F4	min	0	7.65	0	7.09 × 10^−1^	0	0
	std	0	2.81 × 10^4^	0	1.06	0	7.16 × 10^−1^
	avg	0	1.06 × 10^4^	0	3.38	0	3.5 × 10^−1^
	median	0	153.92	0	3.5	0	1.42 × 10^−13^
	worse	0	1.29 × 10^5^	0	5.28	0	2.45
F5	min	0	1.52 × 10^3^	0	27.28	0	2.27 × 10^−13^
	std	0	7.78 × 10^5^	0	857.16	0	81.44
	avg	0	3.94 × 10^5^	0	1.11 × 10^3^	0	17.41
	median	0	1.32 × 10^5^	0	900.4	0	1.18 × 10^−6^
	worse	0	3.93 × 10^6^	0	3.71 × 10^3^	0	446.14
F6	min	0	48.19	0	1.06	0	5.6 × 10^−5^
	std	0	265.02	0	47.05	0	35.43
	avg	0	385.15	0	52.27	0	19.64
	median	0	369.87	0	29.37	0	6.29
	worse	0	1.04 × 10^3^	0	149.01	0	136.41
F7	min	0	1.54 × 10^3^	0	34.55	0	1.1 × 10^−10^
	std	0	2.61 × 10^5^	0	365.05	0	6.5
	avg	0	1.07 × 10^5^	0	614.08	0	1.89
	median	0	2.49 × 10^4^	0	542.68	0	8.42 × 10^−5^
	worse	0	1.36 × 10^6^	0	1.66 × 10^3^	0	33.37
F8	min	0	185.09	0	35.06	0	6.65 × 10^−14^
	std	0	561.57	0	382.66	0	8.12
	avg	0	1.51 × 10^3^	0	553.59	0	2.63
	median	0	1.69 × 10^3^	0	535.58	0	2.43 × 10^−9^
	worse	0	2.35 × 10^3^	0	1.31 × 10^3^	0	32.33
F9	min	0	50.02	1.41 × 10^−315^	9.48 × 10^−1^	1.84 × 10^−315^	1.5 × 10^−9^
	std	0	102.66	0	3.06	0	2.19 × 10^−4^
	avg	8.21 × 10^−313^	156.73	2.69 × 10^−314^	6.33	1.61 × 10^−313^	7.84 × 10^−5^
	median	0	117.47	7.79 × 10^−315^	6.91	4.83 × 10^−314^	5.21 × 10^−6^
	worse	8.34 × 10^−312^	447.85	2.74 × 10^−313^	11.6	2.04 × 10^−312^	9.79 × 10^−4^
F10	min	0	86.62	1.11 × 10^−315^	50.04	1.11 × 10^−315^	8.08 × 10^−5^
	std	0	142.94	0	8	0	34.61
	avg	3.68 × 10^−315^	180.31	1.13 × 10^−315^	55.1	1.25 × 10^−315^	50.67
	median	0	142.6	1.12 × 10^−315^	51.48	1.17 × 10^−315^	66.14
	worse	9.4 × 10^−314^	810.38	1.25 × 10^−315^	77.69	2.35 × 10^−315^	85.54

**Table 6:** Results of the Wilcoxon rank sum test for the ISFOA ablation experiment.

ID	ISFOA vs. SFOA	ISFOA vs. ISFOA1	ISFOA vs. ISFOA2	ISFOA vs. ISFOA3	ISFOA vs. ISFOA4
F1	+	=	+	=	+
F2	+	=	+	=	+
F3	+	=	+	=	+
F4	+	=	+	=	+
F5	+	=	+	=	+
F6	+	=	+	=	+
F7	+	=	+	=	+
F8	+	=	+	=	+
F9	+	=	+	+	+
F10	+	+	+	+	+
+/=/−	10/0/0	1/9/0	10/0/0	2/8/0	10/0/0

**Table 7:** Results of the test function for CEC2005.

	Index	ISFOA	SFOA	PSO	GWO	SSA	RIME	COA	SABO	DBO
	min	0	824.15	4.56 × 10^−2^	2.15 × 10^−29^	4.9 × 10^−193^	1.06	0	1.14 × 10^−200^	1.28 × 10^−159^
	std	0	1.56 × 10^4^	2.06 × 10^−1^	1.08 × 10^−27^	3.67 × 10^−60^	1.37	0	0	1.66 × 10^−99^
F1	avg	0	2.36 × 10^4^	3.15 × 10^−1^	9.29 × 10^−28^	6.75 × 10^−61^	2.57	0	2.18 × 10^−196^	3.03 × 10^−100^
	median	0	2.48 × 10^4^	2.57 × 10^−1^	5.29 × 10^−28^	5.21 × 10^−75^	2.3	0	3.92 × 10^−198^	1.61 × 10^−137^
	worse	0	5.62 × 10^4^	8.54 × 10^−1^	4.62 × 10^−27^	2.01 × 10^−59^	7.77	0	4.35 × 10^−195^	9.08 × 10^−99^
	min	0	5.45	4.09 × 10^−2^	1.12 × 10^−17^	7.34 × 10^−102^	4.33 × 10^−1^	0	7.47 × 10^−113^	2.41 × 10^−86^
	std	0	7.59 × 10^5^	2.52	8.98 × 10^−17^	1.11 × 10^−24^	5.63 × 10^−1^	0	6.25 × 10^−111^	2.19 × 10^−51^
F2	avg	0	1.39 × 10^5^	7.62 × 10^−1^	1.11 × 10^−16^	2.03 × 10^−25^	1.2	0	3.89 × 10^−111^	4.01 × 10^−52^
	median	0	31.35	8.96 × 10^−2^	8.98 × 10^−17^	5.3 × 10^−37^	1.07	0	1.2 × 10^−111^	2.45 × 10^−69^
	worse	0	4.16 × 10^6^	10.05	3.41 × 10^−16^	6.09 × 10^−24^	2.67	0	2.34 × 10^−110^	1.2 × 10^−50^
	min	0	9.58 × 10^3^	565.8	1.25 × 10^−8^	0	644.15	0	1.65 × 10^−82^	1.04 × 10^−156^
	std	0	3.99 × 10^4^	1.02 × 10^3^	4.62 × 10^−5^	7.15 × 10^−24^	511.57	0	1.33 × 10^−43^	7.15 × 10^−55^
F3	avg	0	5.72 × 10^4^	1.69 × 10^3^	1.45 × 10^−5^	1.3 × 10^−24^	1.29 × 10^3^	0	2.43 × 10^−44^	1.31 × 10^−55^
	median	0	4.72 × 10^4^	1.54 × 10^3^	1.39 × 10^−6^	1.25 × 10^−38^	1.08 × 10^3^	0	2.46 × 10^−57^	6.99 × 10^−110^
	worse	0	1.85 × 10^5^	6.31 × 10^3^	2.48 × 10^−4^	3.91 × 10^−23^	2.65 × 10^3^	0	7.3 × 10^−43^	3.92 × 10^−54^
	min	0	26.4	6.01	1.29 × 10^−7^	0	2.65	0	7.26 × 10^−79^	1.34 × 10^−76^
	std	0	17.53	1.1	9.48 × 10^−7^	6.5 × 10^−28^	3.21	0	3.85 × 10^−77^	2.56 × 10^−51^
F4	avg	0	65.32	7.52	1.06 × 10^−6^	1.5 × 10^−28^	7.21	0	3 × 10^−77^	8.84 × 10^−52^
	median	0	65.15	7.6	6.5 × 10^−7^	2.27 × 10^−42^	6.34	0	1.7 × 10^−77^	1.9 × 10^−65^
	worse	0	91.48	10.48	3.92 × 10^−6^	3.51 × 10^−27^	14.99	0	2.06 × 10^−76^	1.12 × 10^−50^
	min	28.46	4.71 × 10^3^	33.63	25.7	2.89 × 10^−9^	53.12	25.61	27.83	25.46
	std	1.16 × 10^−1^	2.84 × 10^7^	428.35	9.82 × 10^−1^	8.31 × 10^−5^	377.87	7.36 × 10^−1^	3.6 × 10^−1^	3.19 × 10^−1^
F5	avg	28.81	2 × 10^7^	331.26	27.07	3.61 × 10^−5^	297.54	26.69	28.38	25.85
	median	28.85	0	150.79	27.12	2.22 × 10^−6^	175.37	26.52	28.57	25.77
	worse	28.92	1.09 × 10^8^	1.88 × 10^3^	28.76	3.74 × 10^−4^	2.03 × 10^3^	28.43	28.84	26.62
	min	2.41	1.66 × 10^3^	5.26 × 10^−2^	8.95 × 10^−5^	6.68 × 10^−16^	7.49 × 10^−1^	7.05 × 10^−3^	1.46	3.06 × 10^−6^
	std	7.87 × 10^−1^	1.23 × 10^4^	1.97 × 10^−1^	3.73 × 10^−1^	1.17 × 10^−11^	7.5 × 10^−1^	3.11 × 10^−1^	5.35 × 10^−1^	2.08 × 10^−3^
F6	avg	4.14	2.01 × 10^4^	2.38 × 10^−1^	7 × 10^−1^	5.21 × 10^−12^	1.94	4.61 × 10^−1^	2.75	1.37 × 10^−3^
	median	4.14	1.77 × 10^4^	1.9 × 10^−1^	5.91 × 10^−1^	4.31 × 10^−13^	1.95	4.47 × 10^−1^	2.75	3.24 × 10^−4^
	worse	5.67	4.73 × 10^4^	9.36 × 10^−1^	1.5	4.28 × 10^−11^	3.55	1.39	3.92	7.1 × 10^−3^
	min	1.88 × 10^−6^	1.02 × 10^−2^	1.75 × 10^−2^	5.24 × 10^−4^	1.44 × 10^−4^	1.34 × 10^−2^	1.4 × 10^−6^	1.05 × 10^−5^	1.35 × 10^−4^
	std	1.18 × 10^−4^	8.46 × 10^−2^	1.47 × 10^−2^	1.48 × 10^−3^	1.91 × 10^−3^	1.36 × 10^−2^	6.66 × 10^−5^	8.46 × 10^−5^	1.44 × 10^−3^
F7	avg	1.58 × 10^−4^	7.65 × 10^−2^	4.38 × 10^−2^	2.28 × 10^−3^	2.02 × 10^−3^	3.91 × 10^−2^	7.29 × 10^−5^	1.13 × 10^−4^	1.55 × 10^−3^
	median	1.52 × 10^−4^	4.77 × 10^−2^	4.17 × 10^−2^	1.96 × 10^−3^	1.55 × 10^−3^	3.9 × 10^−2^	5.43 × 10^−5^	8.65 × 10^−5^	1.12 × 10^−3^
	worse	5.45 × 10^−4^	3.64 × 10^−1^	7.16 × 10^−2^	7 × 10^−3^	9.71 × 10^−3^	6.84 × 10^−2^	3.16 × 10^−4^	3.66 × 10^−4^	6.03 × 10^−3^
	min	−1.04 × 10^4^	−4.99 × 10^3^	−8.95 × 10^3^	−7.67 × 10^3^	−9.85 × 10^3^	−1.09 × 10^4^	−1 × 10^4^	−4.16 × 10^3^	−1.25 × 10^4^
	std	797.26	432.42	548.86	1.07 × 10^3^	583.12	415.07	866.36	407.54	1.7 × 10^3^
F8	avg	−9 × 10^3^	−3.81 × 10^3^	−7.49 × 10^3^	−5.92 × 10^3^	−8.56 × 10^3^	−1.01 × 10^4^	−8.56 × 10^3^	−3.15 × 10^3^	−8.13 × 10^3^
	median	−8.97 × 10^3^	−3.76 × 10^3^	−7.59 × 10^3^	−6 × 10^3^	−8.56 × 10^3^	−1.01 × 10^4^	−8.64 × 10^3^	−3.13 × 10^3^	−7.42 × 10^3^
	worse	−7.5 × 10^3^	−3.25 × 10^3^	−6.64 × 10^3^	−3.27 × 10^3^	−7.47 × 10^3^	−9.09 × 10^3^	−6.02 × 10^3^	−2.33 × 10^3^	−6.24 × 10^3^
	min	0	82.12	26.35	5.68 × 10^−14^	0	29.43	0	0	0
	std	0	38.91	16.67	4.56	0	12.99	0	0	14.17
F9	avg	0	163.37	55.15	3.16	0	60.1	0	0	2.59
	median	0	162.96	56.47	5 × 10^−1^	0	58.73	0	0	0
	worse	0	241.73	94.97	17.9	0	84.88	0	0	77.61
	min	4.44 × 10^−16^	11.76	5.11 × 10^−2^	7.86 × 10^−14^	4.44 × 10^−16^	4.42 × 10^−1^	4.44 × 10^−16^	4 × 10^−15^	4.44 × 10^−16^
	std	0	2.25	6.36 × 10^−1^	1.26 × 10^−14^	0	5.96 × 10^−1^	0	0	0
F10	avg	4.44 × 10^−16^	18.37	7.04 × 10^−1^	1.03 × 10^−13^	4.44 × 10^−16^	2.06	4.44 × 10^−16^	4 × 10^−15^	4.44 × 10^−16^
	median	4.44 × 10^−16^	19.71	4.12 × 10^−1^	1.02 × 10^−13^	4.44 × 10^−16^	2.2	4.44 × 10^−16^	4 × 10^−15^	4.44 × 10^−16^
	worse	4.44 × 10^−16^	19.96	2.26	1.46 × 10^−13^	4.44 × 10^−16^	2.75	4.44 × 10^−16^	4 × 10^−15^	4.44 × 10^−16^
	min	0	48.78	8.68 × 10^−2^	0	0	7.78 × 10^−1^	0	0	0
	std	0	165.15	1.8 × 10^−1^	6.88 × 10^−3^	0	6.44 × 10^−2^	0	0	1.79 × 10^−2^
F11	avg	0	386.55	3.27 × 10^−1^	3.08 × 10^−3^	0	9.59 × 10^−1^	0	0	3.27 × 10^−3^
	median	0	389.94	2.85 × 10^−1^	0	0	9.75 × 10^−1^	0	0	0
	worse	0	660.21	7.31 × 10^−1^	2.82 × 10^−2^	0	1.04	0	0	9.81 × 10^−2^
	min	1.53 × 10^−1^	6.6	4.06 × 10^−4^	1.97 × 10^−2^	9.41 × 10^−16^	3.33 × 10^−1^	1.52 × 10^−4^	9.98 × 10^−2^	1.93 × 10^−7^
	std	2.62 × 10^−1^	1.8 × 10^8^	6.38 × 10^−1^	1.94 × 10^−2^	2.24 × 10^−12^	1.74	7.27 × 10^−3^	1.3 × 10^−1^	3.4 × 10^−4^
F12	avg	5.02 × 10^−1^	9.72 × 10^7^	5.04 × 10^−1^	4.54 × 10^−2^	1.2 × 10^−12^	3.13	1.12 × 10^−2^	2.53 × 10^−1^	8.86 × 10^−5^
	median	4.28 × 10^−1^	9.77 × 10^6^	2.54 × 10^−1^	4.15 × 10^−2^	5 × 10^−14^	2.81	9.79 × 10^−3^	2.38 × 10^−1^	6.47 × 10^−6^
	worse	1.15	5.44 × 10^8^	2.83	9.23 × 10^−2^	7.76 × 10^−12^	7.25	2.89 × 10^−2^	8.36 × 10^−1^	1.87 × 10^−3^
	min	1.4	53.23	2.38 × 10^−2^	9.85 × 10^−2^	1.36 × 10^−15^	1.06 × 10^−1^	1.29	1.85	1.11 × 10^−2^
	std	4.24 × 10^−1^	2 × 10^8^	6.28 × 10^−1^	2.42 × 10^−1^	3.31 × 10^−11^	1.07 × 10^−1^	3 × 10^−1^	2.89 × 10^−1^	5.31 × 10^−1^
F13	avg	2.31	1.31 × 10^8^	4.43 × 10^−1^	5.45 × 10^−1^	1.61 × 10^−11^	2.42 × 10^−1^	2.2	2.87	5.72 × 10^−1^
	median	2.34	5.11 × 10^7^	2.11 × 10^−1^	5.4 × 10^−1^	2 × 10^−12^	2.35 × 10^−1^	2.23	2.97	5.02 × 10^−1^
	worse	2.9	7.64 × 10^8^	3.16	9.96 × 10^−1^	1.43 × 10^−10^	4.39 × 10^−1^	2.97	3.01	2.37
	min	10 × 10^−1^	9.99 × 10^−1^	10 × 10^−1^	10 × 10^−1^	10 × 10^−1^	10 × 10^−1^	10 × 10^−1^	9.99 × 10^−1^	10 × 10^−1^
	std	7.33 × 10^−1^	5.35	7.66 × 10^−1^	4.21	5.65	2.35 × 10^−12^	4.38	2.96	9.28 × 10^−1^
F14	avg	1.26	5.3	1.23	4.56	7.16	10 × 10^−1^	4.52	3.54	1.76
	median	10 × 10^−1^	3.01	10 × 10^−1^	2.98	10.76	10 × 10^−1^	2.98	2.99	10 × 10^−1^
	worse	3.97	21.16	3.97	10.76	12.67	10 × 10^−1^	12.67	12.76	2.98
	min	3.13 × 10^−4^	4.49 × 10^−4^	3.08 × 10^−4^	3.07 × 10^−4^	3.07 × 10^−4^	4.72 × 10^−4^	3.08 × 10^−4^	3.18 × 10^−4^	3.07 × 10^−4^
	std	5.23 × 10^−3^	3.25 × 10^−4^	6.03 × 10^−3^	8.11 × 10^−3^	3.17 × 10^−6^	8.43 × 10^−3^	6.03 × 10^−3^	3.42 × 10^−3^	5.27 × 10^−4^
F15	avg	2.79 × 10^−3^	9.83 × 10^−4^	2.61 × 10^−3^	4.42 × 10^−3^	3.08 × 10^−4^	5.34 × 10^−3^	2.55 × 10^−3^	1.75 × 10^−3^	8.69 × 10^−4^
	median	1.71 × 10^−3^	7.88 × 10^−4^	5.88 × 10^−4^	4.17 × 10^−4^	3.07 × 10^−4^	7.16 × 10^−4^	5.78 × 10^−4^	5.18 × 10^−4^	7.22 × 10^−4^
	worse	2.19 × 10^−2^	1.68 × 10^−3^	2.04 × 10^−2^	2.04 × 10^−2^	3.2 × 10^−4^	2.04 × 10^−2^	2.04 × 10^−2^	1.41 × 10^−2^	2.25 × 10^−3^
	min	−1.03	−1.03	−1.03	−1.03	−1.03	−1.03	−1.03	−1.03	−1.03
	std	1.8 × 10^−4^	2.05 × 10^−5^	6.25 × 10^−16^	2.91 × 10^−8^	5.53 × 10^−16^	3.83 × 10^−7^	9.76 × 10^−16^	1.03 × 10^−2^	5.9 × 10^−16^
F16	avg	−1.03	−1.03	−1.03	−1.03	−1.03	−1.03	−1.03	−1.02	−1.03
	median	−1.03	−1.03	−1.03	−1.03	−1.03	−1.03	−1.03	−1.03	−1.03
	worse	−1.03	−1.03	−1.03	−1.03	−1.03	−1.03	−1.03	−9.97 × 10^−1^	−1.03
	min	3.98 × 10^−1^	3.98 × 10^−1^	3.98 × 10^−1^	3.98 × 10^−1^	3.98 × 10^−1^	3.98 × 10^−1^	3.98 × 10^−1^	3.98 × 10^−1^	3.98 × 10^−1^
	std	4.1 × 10^−7^	1.43 × 10^−3^	0	2.45 × 10^−6^	0	3.31 × 10^−7^	3.74 × 10^−9^	9.91 × 10^−2^	0
F17	avg	3.98 × 10^−1^	3.99 × 10^−1^	3.98 × 10^−1^	3.98 × 10^−1^	3.98 × 10^−1^	3.98 × 10^−1^	3.98 × 10^−1^	4.54 × 10^−1^	3.98 × 10^−1^
	median	3.98 × 10^−1^	3.98 × 10^−1^	3.98 × 10^−1^	3.98 × 10^−1^	3.98 × 10^−1^	3.98 × 10^−1^	3.98 × 10^−1^	4.09 × 10^−1^	3.98 × 10^−1^
	worse	3.98 × 10^−1^	4.04 × 10^−1^	3.98 × 10^−1^	3.98 × 10^−1^	3.98 × 10^−1^	3.98 × 10^−1^	3.98 × 10^−1^	7.71 × 10^−1^	3.98 × 10^−1^
	min	3	3	3	3	3	3	3	3	3
	std	4.49 × 10^−6^	1.2 × 10^−4^	1.47 × 10^−15^	4.98 × 10^−5^	4.93	14.79	2.54 × 10^−11^	9.03 × 10^−1^	4.93
F18	avg	3	3	3	3	3.9	5.7	3	3.55	3.9
	median	3	3	3	3	3	3	3	3.12	3
	worse	3	3	3	3	30	84	3	6.68	30
	min	−3.86	−3.86	−3.86	−3.86	−3.86	−3.86	−3.86	−3.86	−3.86
	std	4.29 × 10^−2^	1.78 × 10^−3^	1.41 × 10^−1^	2.15 × 10^−3^	2.28 × 10^−15^	2 × 10^−7^	2.17 × 10^−12^	1.94 × 10^−1^	3.21 × 10^−3^
F19	avg	−3.84	−3.86	−3.84	−3.86	−3.86	−3.86	−3.86	−3.6	−3.86
	median	−3.86	−3.86	−3.86	−3.86	−3.86	−3.86	−3.86	−3.64	−3.86
	worse	−3.64	−3.85	−3.09	−3.85	−3.86	−3.86	−3.86	−2.86	−3.85
	min	−3.32	−3.32	−3.32	−3.32	−3.32	−3.32	−3.32	−3.32	−3.32
	std	9.99 × 10^−2^	6.17 × 10^−2^	6.97 × 10^−2^	7 × 10^−2^	5.92 × 10^−2^	5.92 × 10^−2^	6.81 × 10^−2^	1.72 × 10^−1^	8.64 × 10^−2^
F20	avg	−3.14	−3.26	−3.26	−3.28	−3.27	−3.27	−3.27	−3.18	−3.24
	median	−3.14	−3.31	−3.26	−3.32	−3.32	−3.32	−3.32	−3.24	−3.2
	worse	−2.97	−3.17	−3.14	−3.14	−3.2	−3.2	−3.08	−2.67	−3.09
	min	−10.15	−10.05	−10.15	−10.15	−10.15	−10.15	−10.15	−6.98	−10.15
	std	1.02	2.72	3.55	2.37	2.29	2.7	2.69	4.43 × 10^−1^	2.67
F21	avg	−9.87	−4.43	−5.66	−8.89	−8.79	−7.7	−7.87	−5.09	−7.19
	median	−10.15	−2.65	−2.68	−10.15	−10.15	−10.15	−10.15	−5.05	−5.1
	worse	−4.96	−1.91	−2.63	−2.63	−5.06	−2.63	−2.68	−3.81	−2.63
	min	−10.4	−10.13	−10.4	−10.4	−10.4	−10.4	−10.4	−5.95	−10.4
	std	9.87 × 10^−1^	2.76	3.61	1.03 × 10^−3^	2.68	3.32	2.7	2.66 × 10^−1^	2.63
F22	avg	−10.15	−6.67	−5.83	−10.4	−8.1	−7.77	−7.75	−5.1	−8.29
	median	−10.4	−7.27	−3.25	−10.4	−10.4	−10.4	−7.75	−5.08	−10.4
	worse	−5.07	−2.66	−2.75	−10.4	−5.09	−2.75	−5.09	−4.58	−5.08
	min	−10.54	−10.41	−10.54	−10.54	−10.54	−10.54	−10.54	−9.27	−10.54
	std	7.05 × 10^−1^	3.02	3.74	1.02 × 10^−3^	2.52	2.5	2.79	1.29	2.69
F23	avg	−10.35	−5.01	−7.8	−10.54	−8.8	−9.06	−7.61	−5.14	−8.83
	median	−10.54	−4.23	−10.54	−10.54	−10.54	−10.54	−5.13	−4.94	−10.53
	worse	−6.93	−1.67	−1.86	−10.53	−5.13	−3.84	−3.84	−3.68	−2.43

**Table 8:** Results of the Wilcoxon rank-sum test for ISFOA on the CEC2005.

ID	SFOA	PSO	GWO	SSA	RIME	COA	SABO	DBO
F1	+	+	+	+	+	=	+	+
F2	+	+	+	+	+	=	+	+
F3	+	+	+	+	+	=	+	+
F4	+	+	+	+	+	=	+	+
F5	+	+	+	+	+	+	+	+
F6	+	+	+	+	+	+	+	+
F7	+	+	+	+	+	+	=	-
F8	+	+	+	+	+	+	+	+
F9	+	+	+	+	+	=	+	+
F10	+	+	+	+	+	=	+	+
F11	+	+	+	+	+	=	+	+
F12	+	+	+	+	+	+	+	+
F13	+	+	+	+	+	+	+	+
F14	+	+	+	+	+	+	+	+
F15	+	+	+	+	+	+	+	+
F16	+	+	+	+	+	+	+	+
F17	+	+	+	+	+	+	+	+
F18	+	+	+	+	+	+	+	+
F19	+	+	+	+	+	+	+	+
F20	+	+	+	+	+	+	+	+
F21	+	+	+	+	+	+	+	+
F22	+	+	+	+	+	+	+	+
F23	+	+	+	+	+	+	+	+
+/=/−	23/0/0	23/0/0	23/0/0	23/0/0	23/0/0	16/7/0	22/1/0	22/0/1

**Table 9:** Results of the Test Function for CEC2021.

ID	Index	ISFOA	SFOA	PSO	GWO	SSA	RIME	COA	SABO	DBO
F1	min	0	6.01 × 10^7^	2.1 × 10^−6^	1.25 × 10^−55^	8.87 × 10^−242^	1.12 × 10^3^	0	1.88 × 10^−199^	3.28 × 10^−156^
	std	0	3.03 × 10^9^	2.54 × 10^3^	1.81 × 10^−51^	7.33 × 10^−35^	7.74 × 10^3^	0	0	4.12 × 10^−87^
	avg	0	2.47 × 10^9^	666.67	6.62 × 10^−52^	1.34 × 10^−35^	1.22 × 10^4^	0	2.2 × 10^−194^	7.52 × 10^−88^
	median	0	1.04 × 10^9^	9.97 × 10^−5^	1.43 × 10^−52^	1.98 × 10^−48^	1 × 10^4^	0	1.15 × 10^−195^	1.4 × 10^−132^
	worse	0	1.02 × 10^10^	1 × 10^4^	9.8 × 10^−51^	4.01 × 10^−34^	3.66 × 10^4^	0	2.74 × 10^−193^	2.25 × 10^−86^
F2	min	0	907.52	3.12 × 10^−1^	9.09 × 10^−13^	0	3.87	0	0	0
	std	0	491.8	150.32	31.75	0	96.68	0	8.66 × 10^−1^	224.76
	avg	0	1.9 × 10^3^	116.43	9.85	0	136.16	0	1.58 × 10^−1^	83.28
	median	0	1.96 × 10^3^	13.6	1.65 × 10^−1^	0	128.76	0	0	0
	worse	0	2.63 × 10^3^	480.65	128.24	0	369	0	4.74	906.28
F3	min	0	55.11	3.61	0	0	1.05	0	0	0
	std	0	90.53	4.96	19.03	0	3.87	0	0	20.3
	avg	0	190.77	12.96	32.95	0	11.79	0	0	22.35
	median	0	168.36	14.7	33.55	0	12.83	0	0	24.86
	worse	0	436.99	20.3	62.36	0	16.26	0	0	52.45
F4	min	0	3.68	4.39 × 10^−1^	0	0	1.74 × 10^−1^	0	0	0
	std	0	1.13 × 10^4^	4.72 × 10^−1^	7.79 × 10^−1^	0	4.51 × 10^−1^	0	0	9.54 × 10^−1^
	avg	0	3.76 × 10^3^	1.08	8.1 × 10^−1^	0	1.1	0	0	7.01 × 10^−1^
	median	0	87.97	1.01	6.11 × 10^−1^	0	9.72 × 10^−1^	0	0	0
	worse	0	5.28 × 10^4^	2.22	2.54	0	2.43	0	0	2.9
F5	min	0	1.54 × 10^3^	9.95 × 10^−1^	6.25 × 10^−24^	1.51 × 10^−140^	4.74	0	4.47 × 10^−23^	2.69 × 10^−78^
	std	0	7.62 × 10^5^	285.05	1.21	8.22 × 10^−16^	84.54	0	1.69 × 10^−21^	2.2 × 10^−1^
	avg	0	4.62 × 10^5^	182.5	7.31 × 10^−1^	4.11 × 10^−16^	87.01	0	1.18 × 10^−21^	4.01 × 10^−2^
	median	0	1.34 × 10^5^	61.7	1.42 × 10^−19^	1.39 × 10^−17^	34.11	0	5.62 × 10^−22^	1.52 × 10^−22^
	worse	0	2.99 × 10^6^	1.08 × 10^3^	4.23	3.09 × 10^−15^	265.27	0	7.11 × 10^−21^	1.2
F6	min	0	39.54	2.59 × 10^−1^	2.43 × 10^−2^	0	4 × 10^−1^	0	4.3 × 10^−6^	0
	std	0	240.16	34.69	1.03	5.56 × 10^−5^	34.95	0	35.68	3.21
	avg	0	333.23	21.03	7.7 × 10^−1^	1.25 × 10^−5^	17.76	0	11.43	8.77 × 10^−1^
	median	0	255.4	11.69	3.27 × 10^−1^	1.78 × 10^−7^	3.81	0	1.31 × 10^−4^	6.47 × 10^−5^
	worse	0	1.03 × 10^3^	120.02	4.35	3.06 × 10^−4^	119.79	0	173.59	12.73
F7	min	0	714.88	2.91 × 10^−1^	6.51 × 10^−4^	0	9.56 × 10^−1^	0	1.1 × 10^−5^	4.47 × 10^−55^
	std	0	9.53 × 10^4^	193.2	1.14	6.17 × 10^−3^	51.18	0	1.07 × 10^−4^	7.15 × 10^−2^
	avg	0	5.36 × 10^4^	103.48	4.12 × 10^−1^	1.38 × 10^−3^	35.24	0	8.82 × 10^−5^	1.77 × 10^−2^
	median	0	1.29 × 10^4^	21.54	2.57 × 10^−2^	7.51 × 10^−7^	6.98	0	4.7 × 10^−5^	1.59 × 10^−5^
	worse	0	4.25 × 10^5^	842.44	6.08	3.36 × 10^−2^	138.51	0	5.16 × 10^−4^	3.79 × 10^−1^
F8	min	0	557.03	1.71 × 10^−7^	0	0	2.07 × 10^−1^	0	0	0
	std	0	611.03	47.53	0	6.76 × 10^−17^	182.12	0	0	0
	avg	0	1.69 × 10^3^	34.7	0	1.23 × 10^−17^	128.75	0	0	0
	median	0	1.66 × 10^3^	30.7	0	0	26.76	0	0	0
	worse	0	2.8 × 10^3^	278.61	0	3.7 × 10^−16^	538.45	0	0	0
F9	min	0	44.83	2.24 × 10^−5^	8.88 × 10^−15^	0	1.52 × 10^−1^	1.4 × 10^−315^	4.38 × 10^−33^	6.16 × 10^−167^
	std	0	105.54	8.51 × 10^−1^	4.26 × 10^−15^	2.48 × 10^−45^	2.11	0	3.82 × 10^−15^	2.08 × 10^−105^
	avg	8.07 × 10^−313^	139.09	1.55 × 10^−1^	1.18 × 10^−14^	4.53 × 10^−46^	1.94	1.55 × 10^−308^	6.81 × 10^−15^	3.79 × 10^−106^
	median	0	102.45	4.96 × 10^−5^	8.88 × 10^−15^	1.96 × 10^−64^	1.13	1.81 × 10^−311^	8.88 × 10^−15^	2.22 × 10^−142^
	worse	1.2 × 10^−311^	573.41	4.66	1.78 × 10^−14^	1.36 × 10^−44^	8.6	3.65 × 10^−307^	8.88 × 10^−15^	1.14 × 10^−104^
F10	min	0	62.03	48.1	3.31 × 10^−3^	0	48.22	1.11 × 10^−315^	6.19 × 10^−4^	7.11 × 10^−15^
	std	0	172.87	9.85	19.05	1.78 × 10^−4^	13.56	0	2.56 × 10^−4^	28.18
	avg	6.17 × 10^−316^	191.54	52.36	54.42	1.23 × 10^−4^	56.28	1.33 × 10^−308^	1.04 × 10^−3^	25.37
	median	0	124.06	48.39	51	5.51 × 10^−5^	48.86	4.97 × 10^−315^	1 × 10^−3^	2.96 × 10^−3^
	worse	9.14 × 10^−315^	907.98	81.66	79.4	6.34 × 10^−4^	82.81	3.23 × 10^−307^	1.82 × 10^−3^	82.43

**Table 10:** Results of the Wilcoxon rank-sum test for ISFOA on the CEC2021.

ID	SFOA	PSO	GWO	SSA	RIME	COA	SABO	DBO
F1	+	+	+	+	+	=	+	+
F2	+	+	+	=	+	=	+	+
F3	+	+	+	=	+	=	=	+
F4	+	+	+	=	+	=	=	+
F5	+	+	+	+	+	=	+	+
F6	+	+	+	+	+	=	+	+
F7	+	+	+	+	+	=	+	+
F8	+	+	=	=	+	=	=	=
F9	+	+	+	+	+	+	+	+
F10	+	+	+	+	+	+	+	+
+/=/−	10/0/0	10/0/0	9/1/0	6/4/0	10/0/0	2/8/0	7/3/0	9/1/0

**Table 11:** The real-world constrained engineering optimization problems.

Problem	Name	D
P1	Tension/compression spring design problem	3
P2	Pressure vessel design problem	4
P3	Three-bar truss design problem	2
P4	Welded beam design problem	4
P5	Speed reducer design problem	7
P6	Cantilever beam design problem	5
P7	Step-cone pulley problem	5

**Table 12:** Test results of engineering problems.

ID	Index	ISFOA	SFOA	PSO	GWO	SSA	RIME	COA	SABO	DBO
P1	best	1.27 × 10^−2^	1.29 × 10^−2^	1.27 × 10^−2^	1.27 × 10^−2^	1.27 × 10^−2^	1.27 × 10^−2^	1.27 × 10^−2^	1.28 × 10^−2^	1.27 × 10^−2^
	std	1.67 × 10^−5^	8.77 × 10^−4^	2.55 × 10^−4^	1.38 × 10^−3^	2.83 × 10^−4^	2.03 × 10^−3^	1.3 × 10^−4^	7.71 × 10^−4^	6.83 × 10^−4^
	mean	1.27 × 10^−2^	1.3 × 10^−2^	1.28 × 10^2^	1.33 × 10^−2^	1.28 × 10^−2^	1.5 × 10^−2^	1.27 × 10^−2^	1.31 × 10^−2^	1.29 × 10^−2^
	rand	1	6	5	7	4	9	3	8	2
P2	min	5.89 × 10^3^	9.39 × 10^3^	5.9 × 10^3^	5.89 × 10^3^	5.89 × 10^3^	5.94 × 10^3^	5.91 × 10^3^	7.18 × 10^3^	5.89 × 10^3^
	std	193.26	1.87 × 10^5^	3.98 × 10^3^	290.57	416.99	492.97	395.28	9.73 × 10^3^	570.38
	mean	6.15 × 10^3^	1.62 × 10^5^	7.18 × 10^3^	5.98 × 10^3^	6.5 × 10^3^	6.8 × 10^3^	6.32 × 10^3^	1.05 × 10^4^	6.5 × 10^3^
	rand	1	9	5	3	4	7	6	8	2
P3	min	263.9	263.9	263.9	263.9	263.9	263.9	263.9	263.9	263.9
	std	7.52 × 10^−5^	2.1 × 10^−2^	5.09 × 10^−1^	1.26 × 10^−3^	1.79 × 10^−3^	1.17 × 10^−1^	6.62 × 10^−4^	1.59 × 10^−1^	1.55 × 10^−4^
	mean	263.9	263.91	264.03	263.9	263.9	263.96	263.9	263.97	263.9
	rand	1	8	7	6	4	5	3	9	2
P4	min	1.69	1.72	1.69	1.69	1.69	1.7	1.69	1.79	1.69
	std	1.12 × 10^−6^	1.92 × 10^−1^	2.74 × 10^−1^	1.11 × 10^−3^	4.72 × 10^−3^	1.18 × 10^−1^	9.04 × 10^−4^	6.29 × 10^−1^	3.22 × 10^−2^
	mean	1.69	1.88	1.82	1.69	1.69	1.78	1.69	2.42	1.69
	rand	1	8	7	5	3	6	4	9	2
P5	min	2.99 × 10^3^	3.05 × 10^3^	3 × 10^3^	3 × 10^3^	2.99 × 10^3^	3 × 10^3^	2.99 × 10^3^	3.22 × 10^3^	2.99 × 10^3^
	std	19.59	70.36	16.19	4.53	3.44 × 10^−4^	2.65	1.53	3.92 × 10^96^	12.16
	mean	3.01 × 10^3^	3.15 × 10^3^	3.01 × 10^3^	3.01 × 10^3^	2.99 × 10^3^	3 × 10^3^	3 × 10^3^	1.7 × 10^96^	3 × 10^3^
	rand	2	8	7	6	3	5	4	9	1
P6	min	1.34	1.87	1.34	1.34	1.34	1.34	1.34	1.4	1.34
	std	1.81 × 10^−6^	1.63	5.94 × 10^−3^	2.73 × 10^−5^	9.27 × 10^−5^	3.74 × 10^−3^	2.05 × 10^−5^	6.64 × 10^−2^	3.91 × 10^−6^
	mean	1.34	4.26	1.35	1.34	1.34	1.35	1.34	1.51	1.34
	rand	1	8	5	4	6	7	3	9	2
P7	min	16.13	8.17 × 10^95^	18.26	3.27 × 10^91^	2.36 × 10^90^	3.87 × 10^88^	1.51 × 10^87^	1.88 × 10^96^	16.15
	std	1.61 × 10^96^	1.88 × 10^97^	1.17 × 10^73^	7.49 × 10^92^	1.11 × 10^92^	1.03 × 10^91^	8.89 × 10^90^	3.43 × 10^97^	3.64 × 10^70^
	mean	2.93 × 10^95^	1.9 × 10^97^	3.93 × 10^72^	1.09 × 10^93^	1.02 × 10^92^	8.71 × 10^90^	3.3 × 10^90^	3.86 × 10^97^	1.31 × 10^70^
	rand	1	8	3	7	6	5	4	9	2

## Data Availability

The code supporting the findings of this study is openly available in the GitHub repository at: https://github.com/Lv1118/algorithm, accessed on 9 January 2026.
